# QuickStats

**Published:** 2015-06-26

**Authors:** FROM THE NATIONAL CENTER FOR HEALTH STATISTICS

**Figure:**
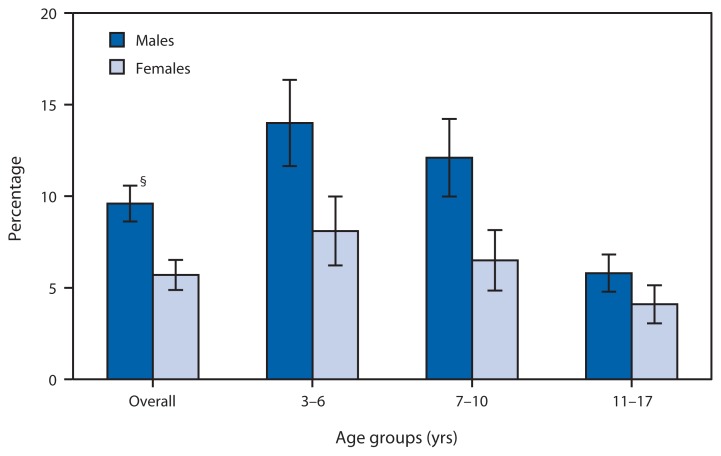
Percentage of Children and Adolescents Aged 3–17 Years with a Reported Communication Disorder During the Previous 12 Months,^*^ by Sex and Age Group — National Health Interview Survey, United States, 2012^†^ ^*^ Based on a positive response from a knowledgeable adult to any of the following four questions regarding a sample child in the household: “During the past 12 months, has [child’s name] had any problems or difficulties that lasted for 1 week or longer with 1) voice, such as too weak, hoarse, or strained; 2) swallowing food or beverages; 3) speaking, such as making speech sounds correctly or stuttering; or 4) learning, using, or understanding words or sentences.” ^†^ Estimates were derived from the National Health Interview Survey sample child component, based on household interviews with a national sample of the civilian, noninstitutionalized U.S. population. ^§^ 95% confidence interval.

During 2012, among children and adolescents aged 3–17 years, males (9.6%) were more likely than females (5.7%) to have had a communication disorder during the previous 12 months; this difference was observed overall and also for each age group (3–6, 7–10, and 11–17 years). The percentage of children and adolescents who had a communication disorder in the previous 12 months declined with increasing age for both males and females.

**Source:** Black LI, Vahratian A, Hoffman HJ. Communication disorders and use of intervention services among children aged 3–17 years: United States, 2012. NCHS data brief, no. 205. Hyattsville, MD: US Department of Health and Human Services, CDC, National Center for Health Statistics; 2015. Available at http://www.cdc.gov/nchs/data/databriefs/db205.pdf.

**Reported by:** Lindsey I. Black, MPH, lblack1@cdc.gov, 301-458-4548; Anjel Vahratian, PhD.

